# *Cuscuta reflexa* and *Carthamus Oxyacantha*: potent sources of alternative and complimentary drug

**DOI:** 10.1186/s40064-015-0854-5

**Published:** 2015-02-11

**Authors:** Muhammad Asam Raza, Fareeha Mukhtar, Muhammad Danish

**Affiliations:** Centre of Natural Products, Department of Chemistry, University of Gujrat, Gujrat, Pakistan

**Keywords:** Medicinal plants, Antioxidant, Antibacterial agents, Enzyme inhibition, Phytochemical screening

## Abstract

The present study was designed to evaluate the biological potential of *Cuscuta reflexa* and *Carthamus oxyacantha*. The ethanolic crude extract (*C.* reflexa; 9.1% and *C. oxyacantha*; 10.4%) was partitioned with different solvents at pH 3.0, 9.0 and 7.0. Phytochemical study showed that n-hexane fractions were rich source of terpenoids and ethyl acetate fractions were phenolic in nature while chloroform fractions contained alkaloidal skeleton. Total phenols were calculated with FC method and ranged 3.5 to 71.4 mg GAE/100 g DW. Antioxidant (DPPH & FRAP), enzyme inhibition potential (Protease & AChE) and antimicrobial activities were examined by the standard protocols. It was observed that about all extracts exhibited significant DPPH activity range (IC_50_ 09 ± 0.5 to 62 ± 1.2 μg/ml). The DPPH active extracts/fractions also showed remarkable reducing potential. A strong correlation has been found between phenolics and antioxidant activities. Antimicrobial assay that was performed against four microbes and results revealed that FMC-6 and FMP-8 were active against all the tested microbes, while FMP-2 was inactive. Eight extracts/fractions of these plants expressed more than 50% inhibition of the targeted enzymes.

## Introduction

The nature’s garden of medicine is being exposed since ancient civilization era. Still more than three fourth (3/4) of the world population depends on medicinal plants or their extracts for life sustaining process. As these are capable to assuage and treat human ailments and infirmity. The metabolites particularly the secondary one (terpenoids, alkaloids, saponins, flavonoids, steroids etc.) that are produced by plants are responsible for the therapeutic outcomes. The remedial plants have extensive application in food industry pharmaceutical, agricultural and cosmetics (Bukhsh et al. 2007; Fellows 1991; Fransworth and Moris 1976; Ahmad et al. 2006).

*Cuscuta reflexa* Roxb. belongs to family *Cuscutaceae* alternate *Convolvulaceae* commonly known as akasbail or amarwala. It is found in Pakistan, India, China, eastern Asia and Afghanistan *C. reflexa* is used as constituent of many medical compositions, which are effective in the treatment of headache, itching, migraine, chronic catarrh, amnesia, epilepsy, expectorates, prolong fever and constipation. It has been examined as anticonvulsant, muscle relaxant, antioxidant, antihypertensive, cardiotonic, antiviral, and antibacterial. Many phytochems like cuscutine, stigmasterol, kaempferol, dulcitol, myricetin and coumarin have been isolated from *C. reflexa* (Sharma et al. 2012; Manish et al. 2012; Anis et al. 2002).

*Carthamus oxyacantha* belongs to family *Asteraceae* found in Pakistan, Afghanistan, India, Iran, Azerbaijan, Iraq and Kyrgyzstan. It is a winter season plant that grows up in the wheat fields (Zadeh et al. 2011; Meshram et al. 2011). *C. oxyacantha* is used for wound healing and anti-inflammatory purposes. It has potential for antimicrobial, antioxidant and antiworm actions. Young leaves of *C. oxyacantha* are utilized as a vegetable, while seeds are used in cooking and it is an alternative for true saffron as a natural food colorant. Two types of oils are produced by this specie: oleic oil and linoleic oil. Two major pigments are found in its flowers: carthamidin and the carthamin. Seeds and flowers have compounds like glycosides, serotonin, flavonoids, and sterols (Souri et al. 2004; Hassan et al. 2010).

Free radicals are the chemical species which contains unpaired electrons. These unpaired electrons make these species very reactive due to which they cause oxidative stress by reacting with other biological compounds. Oxidative stress is involved in the pathogenesis of many ailments, like Parkinson’s and Alzheimer’s disease. It can damage lipids, carbohydrates, proteins, and DNA in the cells (Ratnam et al. 2006). Antioxidants stand for a prime line of protection in opposition to the reactive oxygen species and free radicals. Plants hold a group of phenolic compounds that have the potential to scavenge free radicals and thus act as natural antioxidants. Herbal medicines that have antioxidant potential are being used worldwide for the treatment of various diseases. Medicinal plants are being focused for the extraction of natural antioxidants that can replace synthetic additives because of the toxicological risks associated with synthetic antioxidants (El-Haci et al. 2013; Drummen et al. 2004).

Infectious diseases are one of the principal reasons behind the early deaths throughout the world, killing almost 50,000 people each day. So a great deal of attention is being paid to drugs, resistance to human pathogenic bacteria. The drugs that can restrain the growth of pathogens or may eradicate them are called antibiotics and these must possess minimum lethality to the host cells. Traditionally a lot of remedial plants are being used for the healing of different infectious diseases because they generate a wide range of compounds that are known for therapeutic activities. *e.g.* penicillin (Perez et al. 1990; Kalayou et al. 2012).

Enzymes are the biological catalyst that accelerates the specific biochemical reactions and this specificity is very essential for life to sustain. The factors which affect enzyme activity are enzyme concentration, the amount of specific enzyme substrate, pH of the medium for enzyme activity, the presence of activators and the presence of inhibitors. If any of these factors is not suitable for a particular reaction the activity of enzyme may change. This may lead to low or over activity of enzyme which results a wide category of ailments such as diabetes, Alzeimer’s disease, lysosomal storage disorders, human immunodeficiency virus (HIV) infection and cancer. The chemical substances which affect the activity of enzymes in a specific chemical way are called inhibitors. The inhibitors may be naturally occurring like antipepsin and antitrypsin or they may be synthetic drugs like temocapril, sulfa drugs and lisinopril. The application of these drugs as specific enzyme inhibitors, inhibits the unwanted metabolic pathways in the body and for that reason these drugs are named antimetabolites. The synthetic drugs produce side effects so this research is focused on the search of natural inhibitors for economic and safety purposes. The medicinal plant extracts and plant-derived chemicals can replace this therapeutic approach for the treatment of a wide category of disorders. For example the phenolic compounds, obtained from plants play a significant role in mediating amylase inhibition (Braga et al. 2007; Fan et al. 2010).

During last decades use of herbal medicines is increasing rapidly because of their no side effects, easy access and low cost. Keeping in view the medicinal importance of plants, the present study was carried out to assess the biological activities of these selected plants and qualitative and quantitative estimation of phytochemicals present in these plants.

## Materials and methods

### Chemicals

2,2-Diphenyl-1-picrylhydrazyl (DPPH), Folin-Ciocalteu (FC) reagent, 2,4,6-Tripyridyl-*s*-triazine (TPTZ), 5,5′-Dithio-bis-[2-nitrobenzoic acid] (DTNB), Nα-benzoyl-DL-arginine-paranitroanilide hydrochloride (BApNA), Proteases, Acetylcholine esterase (AChE), Acetylcholine iodide (AChI) were purchased from Sigma-Aldrich/Fluka (Germany). Microbes used in this studies were gifted by Department of Zoology, University of Gujrat, Gujrat (Pakistan).

### Collection of plant material

*Cuscuta reflexa* Roxb. was collected from different areas of the Azad Kashmir while, *Carthamus oxyacantha* was collected from District Chakwal (Punjab). The plants were identified at the Botany Department of University of Gujrat, Gujrat (Pakistan).

### Preparation of plant extracts

The fresh plant material was dried in shade at room temperature for twenty days then grinded. Extraction of pulverized plant material (100 grams each) was carried out by soaking in 5.0 L (ethanol:water; 90:10) for ten days with shaking at regular interval. The extracts were filtered with filter paper and then concentrated it at low temperature and reduced pressure on rotary evaporator to yield residue (crude extract). Crude extract was dissolved in dist. water and after stirring filtered the material, then the filtrate was fractioned with *n*-hexane, chloroform (pH 3, 9 and 7), ethyl acetate and *n*-butanol.

### Phytochemical studies

Phytochemical studies were carried out with the different spraying and coloring agents (ferric chloride, *n*-butanol-HCl, AlCl_3,_ cerric sulphate, urea-HCl, and dragendorff’s) for the detection of the phenolics, triterpenoids, saponins, alkaloids, flavonoids and sugars (Praveen and Sharmishtha 2012; Williams and Grayer 2004).

### Antioxidant activity

#### DPPH assay

For the estimation of the anti-radical potential, DPPH radical scavenging activity of all the extracts/fractions was conducted using DPPH method (Shahwar et al. 2010). To 100 μl of the extract (2 mg/ml) added 2 ml of the DPPH solution (25 mg/l) and kept the mixture for 30 min at room temperature. Methanol was used as a baseline correction and the absorbance of the fractions/samples was observed at 517 nm using UV/Vis Spectrophotometer. Gallic acid was used as standard and the antioxidant activity of all extracts was expressed as percentage inhibition using following formula;

$$ \%\kern0.5em \mathrm{Inhibition}\ \mathrm{of}\ \mathrm{DPPH} = \frac{\mathrm{A}-\mathrm{B}}{\mathrm{A}}\times 100 $$

Where; A = absorbance of blank and B = absorbance of the sample

#### FRAP assay

FRAP assay of extracts/fractions was conceded out by using the method of Shahwar et al. (2012). The FRAP reagent consists of 300 mM acetate buffer of pH 3.6, 20 mM solution FeCl_3_.6H_2_O and 10 mM 2,4,6-tripyridyl-*s*-triazine (TPTZ). 200 μl plant extracts were mixed to FRAP reagent, allowed to stand for six minutes and absorbance was noted at 593 nm.

#### Total phenol

Total phenol contents (TPC) of all the fractions were assessed by using Folin-Ciocalteu (FC) reagent. (Qureshi et al. 2011). To 200 μl of each of the sample solution (2 mg/ml) 100 μl of FC reagent and 200 μl of sodium bicarbonate solution (10%) were added. Then left the mixture for 30 min at room temperature and the absorbance was measured at 765 nm using UV/Vis Spectrophotometer. Solutions having varying concentrations of gallic acid were prepared in order to plot a calibration curve. The amount of total phenolic content of each extract/fractions was articulated as mg/100 g equivalent to gallic acid using the above calibration curve.

### Antimicrobial activity

Antimicrobial activity of all extracts was evaluated according to agar well diffusion method (Shahwar et al. 2009) against four selected microbes (*Bacillus* species AQ-1, *Bacillus* species AQ-2, *Bacillus* species AZ-1, *Bacillus* species AZ-2) isolated from soil. Growth medium (Nutrient broth and agar-agar: 2 grams each in 100 ml) was prepared and poured in the petri dishes, cotton dip in the culture medium was swapped on the surface of solid medium. Four small cavities were made by using a sterilized micro-pipette tip (6 mm). 60 μl of extracts (2 mg/ml) were poured out in the hole with the help of micro-pipette. All the steps involved in the preparation of inoculums and petri dishes were performed in aseptic medium. Culture plates containing bacteria were placed in incubator at 37°C. After 24 hours of incubation the zone of inhibition (mm) was observed on plates.

### Enzyme inhibition activity

#### Protease inhibition assay

Protease inhibition assay was carried out by using the method of Shahwar et al. (2011) Tris buffer (100 mM) of pH 7.5 was prepared by dissolving 12.1 g of Tris (hydroxymethyl)-aminomethane in distilled water and adjusted pH 7.5 with HCl (5 M). 2 mg of trypsin (Sigma-Aldrich) were dissolved in 10 ml of 1.0 mM HCl in order to prepare the stock solution. Nα-benzoyl-DL-arginine-paranitroanilide hydrochloride (BApNA) was dissolved in DMSO (20 mg/ml). Enzyme (50 μl) and 200 μl sample solution (2 mg/ml in DMSO) were incubated at 37°C for 15 minutes. Then added 30 μl of substrate and the final volume of 2.5 ml was made by adding tris buffer. Then again incubated the reaction mixture at 37°C for 30 minutes. The absorbance was noted using UV/Vis spectrophotometer at 410 nm. The percentage inhibition was determined by using the following formula:

$$ \%\;\mathrm{Inhibition}=\frac{\mathrm{Absorbance}\ \left(\mathrm{blank}\right) - \mathrm{Absorbance}\;\left(\mathrm{test}\right)}{\mathrm{Absorbance}\;\left(\mathrm{blank}\right)}\times 100 $$

### Acetylcholine esterase inhibition activity

Acetylcholine esterase inhibition activity of plant extract was evaluated by using the spectrophotometric method explained by Abbasi et al. (2012). 50 μl of each sample solution (2 mg/ml in DMSO) was taken in a test tube, added 50 μl of enzyme and incubated reaction mixture for 15 min at room temperature. After incubation, added 30 μl of DTNB followed by the 30 μl addition of substrate (AChI). Absorbance was noted at 412 nm using UV/Vis spectrophotometer.

## Results and discussion

Health treatments based on medicinal plants are being prescribed by doctors in the form of plant extracts, infusion or by direct ingestion of very fine powder of plant. Likewise these are recommended as a nutritional supplement for the treatment of everyday problems such as stress and insomnia. There is a resurgence of interest in herbal medicine for the treatment of various ailments, chiefly because of the prohibitive cost of allopathic drugs, their unavailability in remote areas and the popular belief that naturally occurring products are without any adverse side-effects.

From a medical point of view, the important constituents of plants are pharmacologically active compounds such as flavonoids, alkaloids, glycosides and similar other organic substances. In addition, medicinal plants contain essential and trace elements, which can be available to the human body on consumption of herbs and their extracts. Indeed today many, if not most, pharmacological classes of drugs include a natural product prototype. The search for pharmacologically active chemicals from plant sources has continued and many compounds have been isolated and introduced into clinical medicine. Modern medicine is now beginning to accept the use of standardized plant extracts. Present study was conducted also to enhance the same knowledge further and is focused to investigate chemical composition including phytochemical and biological studies of *Cuscuta reflexa* Roxb & *Carthamus oxyacantha*, *Cuscuta reflexa* Roxb. and *Carthamus oxyacantha*. These were collected from various regions of the Punjab (Pakistan) on the basis of its medicinal importance from available data. The fresh plant material was extracted in aqueous ethanol and then partitioned with different solvent on polarity basis yielding seven organic soluble fractions.

All extracts/fractions were subjected to different test for screening of phytochemical tests which gave indication of phenolics, triterpenoids and alkaloids compounds (Table 1).

**Table 1:** **Phytochemical studies of selected plants**

**Plant sample**	**Alkaloids**	**Saponins**	**Triterpenoids**	**Sugars**
FMC-1	-	-	+	-
FMC-2	-	-	+	-
FMC-3	+++	-	+++	-
FMC-4	++	-	+	-
FMC-5	-	-	+	-
FMC-6	-	-	++	-
FMC-7	-	-	-	-
FMC-8	-	-	-	-
FMP-1	-	-	+	-
FMP-2	-	-	+	-
FMP-3	-	-	++++	-
FMP-4	++	-	+++	-
FMP-5	-	-	++	-
FMP-6	-	-	++	-
FMP-7	-	-	-	-
FMP-8	-	-	-	-

FMC-1; Crude extract of *C. reflexa*, FMC-2; *n*-hexane fraction of *C. reflexa*, FMC-3; Chloroform (acidic) fraction of *C. reflexa*, FMC-4; Chloroform (basic) fraction of *C. reflexa*, FMC-5; Choloroform (neutral) fraction of *C. reflexa*, FMC-6; Ethyl acetate fraction of *C. reflexa*, FMC-7; *n-*butanol fraction of *C. reflexa*, FMC-8; Water fraction of *C. reflexa*, FMP-1; Crude extract of *C. oxycantha*, FMP-2; *n*-hexane fraction of of *C. oxycantha*, FMP-3; Chloroform (acidic) fraction of *C. oxycantha*, FMP-4; Chloroform (basic) fraction of *C. oxycantha*, FMP-5; Choloroform (neutral) fraction of *C. oxycantha*, FMP-6; Ethyl acetate fraction of *C. oxycantha*, FMP-7; *n-*butanol fraction of *C. oxycantha*,, FMP-8; Water fraction of *C. oxycantha*

- Not active + Slight active ++ Moderate active +++ Significantly active.

Total phenolic contents were calculated using FC methods expressed as gallic acid equivalents and it was concluded that ethanolic, chloroform and ethyl acetate extracts contained considerable amounts of phenolic contents (FMC-1:71.4, FMC-6:54.4 & FMP-3:63.7 GAE/100 g DW). The order of phenolics in extracts for *C. reflexa* is FMC-1 > FMC-5 > FMC-7 > FMC-2 > FMC-6 > FMC-3 > FMC-4 > FMC-8 and similarly for C. *oxyacantha* is; FMP-6 > FMP-3 > FMP-7 > FMP-5 > FMP-1 > FMP-4 > FMP-8 > FMP-2 as shown in Table 2.

**Table 2:** **Total phenolic contents and antioxidant activities of selected plants**

**Code**	^**a**^ **Total phenol**	**DPPH activity**		^**c**^ **FRAP**
		^**b**^ **%Inhibition IC**_**50**_ **(** ***μg/ml*** **)**		
FMC-1	71.4 ± 0.8	92.3 ± 1.8	20 ± 0.6	825.9 ± 21.5
FMC-2	62.2 ± 0.8	93.5 ± 1.0	30 ± 0.7	492.5 ± 10.3
FMC-3	53.5 ± 1.1	92.5 ± 0.9	18 ± 0.5	677.8 ± 11.7
FMC-4	45.0 ± 0.7	93.0 ± 1.5	12 ± 0.6	404.8 ± 9.7
FMC-5	66.4 ± 1.2	93.3 ± 1.0	16 ± 0.3	462.8 ± 10.3
FMC-6	54.4 ± 1.3	90.3 ± 1.2	18 ± 0.4	315.1 ± 7.3
FMC-7	65.5 ± 1.0	94.5 ± 1.5	16 ± 0.5	355.8 ± 9.1
FMC-8	36.1 ± 0.7	82.0 ± 0.9	09 ± 0.5	208.7 ± 1.1
FMP-1	40.1 ± 0.6	91.5 ± 1.4	32 ± 0.8	225.7 ± 2.4
FMP-2	3.5 ± 0.0	25.6 ± 0.5	-	5.9 ± 1.0
FMP-3	63.7 ± 0.6	85.6 ± 1.1	24 ± 0.7	623.6 ± 13.5
FMP-4	34.1 ± 0.8	90.2 ± 1.5	40 ± 1.0	431.2 ± 10.3
FMP-5	45.6 ± 0.9	90.0 ± 1.3	58 ± 1.6	511.5 ± 8.1
FMP-6	67.4 ± 0.8	92.2 ± 1.0	32 ± 1.0	606.5 ± 11.1
FMP-7	54.1 ± 1.1	39.1 ± 0.7	-	421.2 ± 7.9
FMP-8	28.7 ± 0.5	52.6 ± 0.5	62 ± 1.2	270.2 ± 6.9

^a^mg GAE/100 g DW ^b^100 µl (2 mg/ml) ^c^200 µl (2 mg/ml) and expressed in μM equivalent to FeSO_4._7H_2_O.

Antioxidant potential of all fractions was monitored with DPPH and FRAP assay. The DPPH is an organic free radical which is very stable with ʎ_max_ 517 nm and it is one of the most commonly used reagents for estimation of antioxidant potential of different compounds. The change of color from purple to yellow as the free radical is hunted by the antioxidants causes a decrease in the absorbance and it was resulted that most of the fractions showed remarkable activity in both assays (Ashokkumar et al. 2008; Conforti et al. 2009). Highest antiradical activity was shown by FMC-7 (94.5%) with IC_50_ 16 μg as shown in Table 2 while the gallic acid was used as standard with 94.4% inhibition and IC_50_ of 4 μg. The maximum reducing power was exhibited by FMC-I = 825.9 μM equivalent to FeSO_4._7H_2_O as shown in Table 3. The findings of the present study revealed that the extracts were free radical inhibitors and acted as primary antioxidants.

**Table 3:** **Enzyme inhibition (%) activity of plant fractions**

**Sample/Fractions**	^**a**^ **Protease**	^**b**^ **AChE**
FMC-1	92.73 ± 1.5	85.42 ± 1.0
FMC-2	82.91 ± 1.1	89.17 ± 08
FMC-3	62.00 ± 1.3	50.81 ± 1.1
FMC-4	15.01 ± 0.7	0.00 ± 0.0
FMC-5	9.41 ± 0.5	0.00 ± 0.0
FMC-6	91.42 ± 1.0	85.22 ± 1.7
FMC-7	5.92 ± 0.5	0.00 ± 0.0
FMC-8	90.40 ± 1.0	91.95 ± 1.5
FMP-1	66.04 ± 0.9	59.01 ± 0.9
FMP-2	0.00 ± 0.0	0.00 ± 0.0
FMP-3	88.05 ± 1.7	83.96 ± 1.1
FMP-4	18.52 ± 0.7	11.50 ± 0.6
FMP-5	0.00 ± 0.0	0.00 ± 0.0
FMP-6	14.91 ± 1.0	9.63 ± 1.0
FMP-7	9.41 ± 0.8	0.00 ± 0.0
FMP-8	96.54 ± 0.9	91.09 ± 1.3

^a^100 μl (2 mg/ml) ^b^50 μl (2 mg/ml).

**Figure 1:**
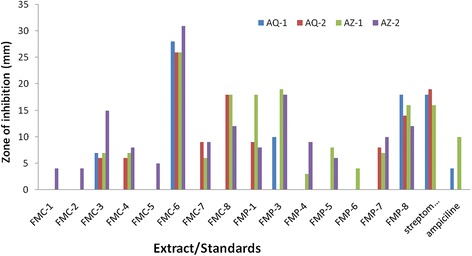
**Antimicrobial activities of extracts against selected** ***Bacillus*** **species.**

Antibacterial activity of all extracts and fractions was done against four soil isolated bacterial strains. FMC-6 and FMP-8 showed a broad spectrum of antimicrobial activity against all the selected bacterium while remaining showed significant activity. It was very interesting that FMC-6 (31 mm against AZ-2, 26 mm against AZ-1, 26 mm against AQ-2 and 28 mm against AQ-1) exhibited activity greater than standards (Streptomycin and Ampiciline) as shown in Figure 1. Plants can supply a wide range of valuable constituents for the development of novel chemotherapeutics. One step towards this objective is the evaluation of *in vitro* antibacterial activity of different plants. Many reports have been presented on the antimicrobial, anti-inflammatory, antimolluscal and antihelmintic potentials of plants. The antibacterial activity has been ascribed due to the existence of some active constituents in the plant extracts. The selected plants exhibited a broad spectrum of antibacterial activities which may be useful for the development of new classes of natural and semi-synthetic antibiotics that might be used to treat a wide category of infectious diseases (Samy and Ignacimuthu 2000; Shahid et al. 2013). So, it is clear from the findings of the current study that the plant extracts have great biological effects. However, there is need to study other biological studies and advance purifications.

Acetylcholine esterase and trypsin (proteases) inhibition studies of plant extracts were conducted using well known reported methods and it was concluded that most of plant extracts showed remarkable enzyme inhibition activity as mentioned in Table 3 particularly by FMC-8 and trypsin inhibition activity by FMP-8. Scientists are engaged in searching of substitute, effective and secure therapeutic agents, a major thrust area in the mainstream of pharmaceutical research. Medicinal plants play a crucial role for the advancement of new drugs. Plant materials remain a significant resource to combat severe diseases in the world. Pharmacognostic investigations of plants are carried out to find new drugs or templates for the development of novel bioactive agents. The use of traditional medicine is expanding globally and their use not only for primary health care in developing countries, but also in countries where conventional medicine is predominant in the national health care system. *In vitro* pharmacological investigations of traditionally used medicinal plants offers an incredible chance to discover and investigate a broad range of plant-based drugs as a potential source of novel biologically active agents and to validate claims made on their safety and efficacy.

## Conclusion

Recent years have seen an exponential increase in research of antioxidant properties of plants and it is accepted that higher intakes of natural antioxidants containing phenolics are associated with long-term health benefits. Free radicals play a significant role in pathogenesis of tissue damage, consequently having implications in many clinical conditions. A lot of research is being under taken to identify new plant resources which have no or minimum side effects and potential antioxidant capacity. So, higher antioxidant potential showed by these plants may prove helpful to treat many diseases like aging, cancer and Parkinson’s disease. The restriction of bacterial growth by the plant extracts in present study validated the conventional use of plants in curing of infections due to which millions of people die each year throughout the world. Bioactive compounds from these plants can therefore be utilize for the formulation of antimicrobial therapeutics for the treatment of a variety of bacterial and fungal infectivity including pneumonia, gonorrhea, mycotic and eye infections. AChE inhibition activities may prove of novel values in clinical trials for the treatment of Alzheimer’s disease. Isolation, identification and purification of these phytochemicals and investigation of their relevant antimicrobial antioxidants, enzyme inhibition potentials and toxicological estimation with the idea of formulating novel chemotherapeutics should be the future direction for searching.
